# Strategy of employing plug-and-play vectors and LC–MS screening to facilitate the discovery of natural products using *Aspergillus oryzae*

**DOI:** 10.1186/s40643-024-00833-w

**Published:** 2025-01-08

**Authors:** Hanliang Shi, Beibei Lin, Mengmeng Zheng, Fengyu Gan, Zhi Lin, Xiujuan Xin, Jian Zhao, Xudong Qu, Faliang An

**Affiliations:** 1 State Key Laboratory of Bioreactor Engineering, East China University of Science and Technology, 130 Mei Long Road, Shanghai, 200237 China; 2 State Key Laboratory of Microbial Metabolism, School of Life Sciences and Biotechnology, Shanghai Jiao Tong University, Shanghai, 200240 China; 3 Key Laboratory of Combinatorial Biosynthesis and Drug Discovery Ministry of Education, School of Pharmaceutical Sciences, Wuhan University, Wuhan, 430071 China; 4 Zhangjiang Institute for Advanced Study, Shanghai Jiao Tong University, Shanghai, 201203 China; 5 Department of Applied Biology, East China University of Science and Technology, 130 Mei Long Road, Shanghai, 200237 China; 6 Marine Biomedical Science and Technology Innovation Platform of Lin-Gang Special Area, No. 4, Lane 218, Haiji Sixth Road, Shanghai, 201306 China

**Keywords:** Plug-and-play vectors, LC–MS detection, *Aspergillus oryzae*, Heterologous expression, P*amyB*

## Abstract

*Aspergillus oryzae* is a widely used host for heterologous expression of fungal natural products. However, the vectors previously developed are not convenient for use and screening positive transformants by PCR and fermentation is time- and effort-consuming. Hence, three plug-and-play vectors were developed here for multi-gene expression and liquid chromatography mass spectrometry detection was introduced to screen positive transformants. Using *rug* BGC for verification, we demonstrated that the vectors we developed perform well and liquid chromatography mass spectrometry detection is feasible to screen positive transformants. For deleterious gene expression, P*xyrA* rather than P*amyB* was employed. Utilizing the toolkit described here to express natural products, dozen days can be saved.

**Graphical Abstract:**

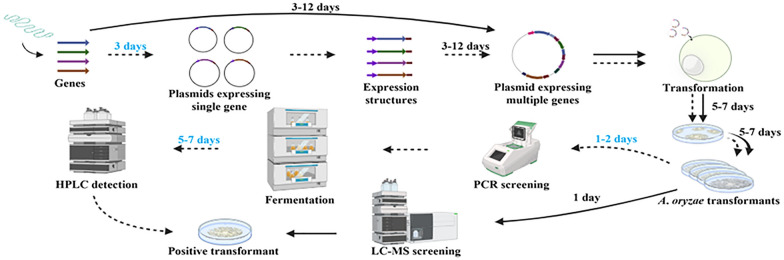

**Supplementary Information:**

The online version contains supplementary material available at 10.1186/s40643-024-00833-w.

## Introduction

As an important reservoir of natural products, fungi have contributed various valuable chemicals such as penicillin and mevastatin (Endo et al. 1976; Fleming 1929). To further excavate novel natural products of fungi for medical and agricultural use, heterologous expression has been undertaken based on genome sequencing and computational tools (Blin et al. 2024; Khaldi et al. 2010; Meng et al. 2022) since most biosynthetic gene clusters (BGCs) responsible for the synthesis of natural products are cryptic under laboratory conditions (Chiang et al. 2009). Historically, unicellular model microbes such as *Escherichia coli* (*E*. *coli*) and yeast, as well as filamentous fungi, were employed to heterologously express BGCs of fungi origin. But nowadays filamentous fungi have moved to center stage with the development of tools for genetic manipulation (Alberti et al. 2017).

**Fig. 1:**
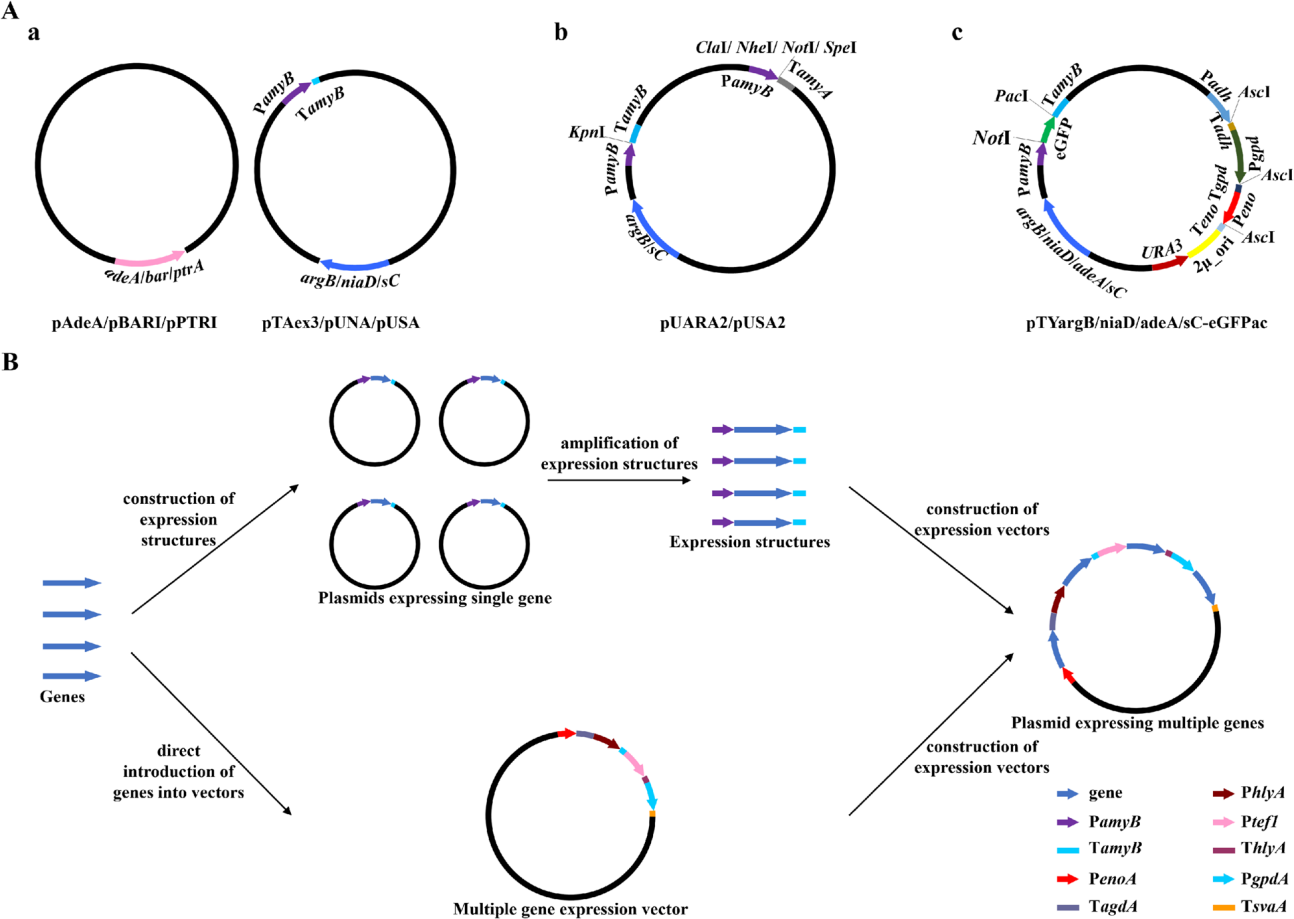
Optimized vectors and the scheme of reconstructing the natural product biosynthetic pathway. **A** Maps of optimized vectors that **a** carry no or one promoter, **b** carry the same promoters, and **c** carry the same restriction sites. **B** The scheme of reconstructing the natural product biosynthetic pathway. When pAdeA, pTAex3, pUSA, pUNA, pBARI and pPTRI are employed to express multiple genes, genes are introduced into pTAex3, pUNA, or pUSA to construct expression structures of promoter-gene-terminator. It usually takes 3 days. And then expression structures are amplified and introduced into the desired vectors to construct plasmids expressing multiple genes. When multiple gene expression vectors developed here are used, the process of construction and amplification of expression structures can be bypassed, and thus 3 days can be saved

*Aspergillus oryzae* (*A*. *oryzae*) is frequently recruited to express fungal BGCs for its clean metabolic background, available genetic manipulation tools, and robust capacity of precursor supply (Awakawa and Abe 2021; Meng et al. 2022). A myriad of valuable natural products from different organisms have been successfully expressed in *A*. *oryzae* such as helvolic acid from *Aspergillus fumigatus* (Lv et al. 2017), erinacines from *Hericium erinaceus* (Liu et al. 2019), and rugulosin A from *Talaromyces* sp. YE3016 (Han et al. 2021). In the process of employing *A*. *oryzae* to express natural products, several *A*. *oryzae* heterologous expression strains have been developed and recruited, such as *A*. *oryzae* M-2-3 (Δ*argB*) (Gomi et al. 1987), *A*. *oryzae* NSAR1 (*niaD*^−^, *sC*^−^, Δ*argB*, *adeA*^−^) (Jin et al. 2004a), and *A*. *oryzae* NSPlD1 (*MAT1-1 niaD*^−^ *sC*^−^ *adeA*^−^ Δ*argB*::*adeA*^−^ Δ*ligD*::*argB* Δ*pyrG*::*adeA*) (Maruyama and Kitamoto 2008). Along with strain optimization, considerable efforts have also been made to optimize vectors for reconstruction of fungal BGCs. The six heterologous expression vectors, pAdeA (Jin et al. 2004b), pTAex3 (Fujii et al. 1995), pUSA (Yamada et al. 2003), pUNA (Yamada et al. 1999), pBARI (Matsuda et al. 2014), and pPTRI (Kubodera et al. 2000), are commonly used to date. Among the six vectors, only pTAex3, pUNA, and pUSA were inserted a P*amyB*-T*amyB* expression cassette (Fig. 1A). It is inconvenient for use since expression structures (promoter-gene-terminator) have to be constructed for subsequent construction of vectors expressing multiple genes. (Fig. 1B). To efficiently reconstruct the whole biosynthetic pathway that comprises multiple genes, several multiple gene expression vectors have been developed (Lazarus et al. 2014; Pahirulzaman et al. 2012; Tagami et al. 2014; Yamane et al. 2017). The common feature of these vectors is that the same promoters or the same restriction sites are employed (Fig. 1A). Tagami et al. (2014) developed pUARA2 and pUSA2 through inserting two copies of P*amyB* (Fig. 1A). But it may create difficulties in reconstructing and expressing biosynthetic pathways. For instance, homologous fragments of the vectors may result in inactivation of genes through undesirable crossovers in the host (Lazarus et al. 2014). Lazarus et al. (2014) constructed pTYargB/niaD/adeA/sC-eGFPac which carry four intact expression cassettes comprising different promoters and terminators. However, the same restriction sites were inserted behind three promoters (Fig. 1A). It means that even if one coding region is assembled, the other two sites have to be destroyed and the resulting unnecessary fragments may reduce the efficiency of recombination. Other optimized vectors, such as pTASU03 (Yamane et al. 2017) and pTAYAGSarg3P (Pahirulzaman et al. 2012), have similar drawbacks. Unlike above studies that focused on improving plasmids to facilitate the reconstruction of BGCs, Yuan et al. (2022) introduced automatic equipment into the work and developed an automated and high-throughput (auto-HTP) biofoundry workflow. With this strategy, they reconstructed 39 BGCs and identified 185 terpenoids. However, the vectors were not equipped with promoter and terminator expression cassettes. In the expression systems introduced above, reconstructed BGCs are randomly integrated into the genome of *A*. *oryzae*. With the heterologous expression strain *A*. *oryzae* NSPlD1 (Maruyama and Kitamoto 2008) and the clustered regularly interspaced short palindromic repeat (CRISPR)-Cas9 system specifically optimized for *A*. *oryzae* (Katayama et al. 2016), Nagamine et al. (2019) and Liu et al. (2019) successfully expressed BGCs of mushroom fungi in a site-specific integration manner. But the vectors carry no promoters such as pXN (Mori et al. 2019), carry one *amyB* promoter such as pDP201a (Liu et al. 2019), or carry the same promoters such as pDP1032 (Nagamine et al. 2019). Therefore, the inconvenience described above may also be encountered in reconstructing and expressing biosynthetic pathways.

Reconstruction of the natural product biosynthetic pathway, transformation of expression vectors into the host, and screening of positive transformants are three primary parts of construction of heterologous expression strains of *A*. *oryzae*. Many efforts have been made to optimize strains and vectors, but little progress has been made in screening positive transformants. Nowadays, primary screening by PCR and re-screening by fermentation are mainly undertaken to screen positive transformants. But it often takes about 7 days.

To facilitate the discovery of natural products using *A*. *oryzae*, the strategy of employing plug-and-play vectors and liquid chromatography mass spectrometry (LC–MS) screening is presented here. Each of the three plug-and-play vectors contains four different promoter-terminator expression cassettes and unique restriction sites were deployed in each promoter and terminator pair. To simplify the screening of positive transformants, constitutive promoters were employed and liquid chromatography mass spectrometry was introduced, making it possible to screen transformants directly on CD agar plates without fermentation in rich medium. Utilizing the strategy described above to express natural products in *A*. *oryzae*, about 10 days could be saved. Additionally, to express deleterious genes, another three vectors were developed in which one or two constitutive promoters were substituted with P*xyrA* (Jeennor et al. 2022) which was superior to P*amyB* in regulating gene expression. It is worth mentioning that the vectors, especially the latter three vectors, are modular, so that expression cassettes could be substituted rapidly and conveniently on demand.

## Materials and methods

### General materials and methods

Primer synthesis and DNA sequencing were performed by TsingKe Biotech Co., Ltd. (Shanghai, China). All the primers used in this study were listed in Table S1. Restriction enzymes, DNA polymerases, and genomic DNA extraction kits were purchased from Yeasen Biotech Co., Ltd. (Shanghai, China). Plasmid purification kits and agarose gel DNA extraction kits were purchased from Sangon Biotech Co., Ltd. (Shanghai, China).

### Strains and media

*E*. *coli* DH10B cultured in LB with carbenicillin supplemented at a final concentration of 50 μg/mL was used for molecular cloning. *Saccharomyces cerevisiae* (*S. cerevisiae*) BJ5464-NpgA (*MATα ura3-52 his3-*∆*200 leu2-*∆*1 trp1 pep4::HIS3 prb1*∆*1.6R can1 GAL*) (Lee et al. 2009) cultured in YPD (1% yeast extract, 2% peptone, 2% glucose) or uracil-minus medium was used for homologous recombination to construct multiple gene expression vectors. *Aspergillus flavus* (*A*. *flavus*) maintained on potato dextrose agar (PDA) was used to amplify P*tef1* and P*gpdA*. P*enoA*, P*hlyA*, and terminators were amplified from *A*. *oryzae* NSAR1 cultivated in DPY medium (2% dextrin, 1% polypeptone, 0.5% yeast extract, 0.05% MgSO_4_·7H_2_O, 0.5% KH_2_PO_4_). Transformants of *A*. *oryzae* were grown on Czapek-Dox (CD) agar plates (21.86% sorbitol, 0.1% K_2_HPO_4_, 0.05% MgSO_4_·7H_2_O, 0.05% KCl, 0.001% FeSO_4_·7H_2_O, 3% sucrose, 2% agar) supplemented with 0.3% (NH_4_)_2_SO_4_, 0.15% methionine, 0.1% arginine, and 0.01% adenine according to the plasmids transformed. MPY medium was used for fermentation in which dextrin of DPY medium was replaced by maltose.

### Construction of multiple gene expression vectors

Promoters and terminators amplified from genomes of *A*. *flavus* and *A*. *oryzae* were ligated as expression cassettes of promoter-unique restriction site-terminator by overlap extension PCR. Unique restriction sites were added by primer synthesis. The amplification and screening cassette of yeast (2μ_ori and uracil synthesis gene) were amplified from pXW55. Then the four expression cassettes, the amplification and screening cassette of yeast, and the *Bam*HI-digested pTAex3 vector were transformed into *S. cerevisiae* BJ5464-NpgA for homologous recombination to obtain the multiple gene expression vector pYEAR227. pYEAA129 and pYEAM46A derived from pAdeA and pUSA were constructed in a similar approach.

### Construction of expression vectors of ***Pc21g16000*** (***pks17***) and ***rug*** gene cluster (Han et al. 2021)

The *pks17* gene was amplified from *Penicillium chrysogenum* (*P*. *chrysogenum*) genomic DNA and it was ligated into the *Eco*RI-digested pYEAR227, the *Kpn*I/*Not*I-digested pYEAR227, the *Spe*I-digested pYEAR227, and the *Nhe*I-digested pYEAR227 to yield pYEAR227-P*enoA*-*pks17*, pYEAR227-P*hlyA*-*pks17*, pYEAR227-P*tef1*-*pks17*, and pYEAR227-P*gpdA*-*pks17*.

To reconstruct the *rug* BGC, the six genes of the BGC were amplified from *Talaromyces* sp. YE3016 kindly provided by Tan (Han et al. 2021). The *rugA* gene was ligated into the *Spe*I-linearized pYEAR227 using Hieff Clone® Plus One Step Cloning Kit to obtain pYEAR227-*rugA*. Then the *rugB* gene was ligated into the *Nhe*I-digested pYEAR227-*rugA* to yield pYEAR227-*rugAB*. In a similar approach, the other four genes of the *rug* BGC was introduced into pYEAM46A to yield pYEAM46A-*rugEFGH* in which *rugE*, *rugF*, *rugG*, and *rugH* were regulated by P*hlyA*, P*gpdA*, P*enoA*, and P*tef1*.

To find a promoter that strictly controls gene expression, the fragment of *pks17*-T*hlyA* was amplified from pYEAR227-P*tef1*-*pks17* and ligated into the *Pst*I-linearize pAdeA to yield pAdeA-*pks17*. Then, P*amyB* and P*xyrA* amplified from *A*. *oryzae* genomic DNA were introduced into the *Swa*I-digested pAdeA-*pks17* to yield pAdeA-P*amyB*-*pks17*, pAdeA-P*xyrA*-*pks17*.

### Transformation of *S*. *cerevisiae* BJ5464-NpgA and *A*. *oryzae* NSAR1

Frozen-EZ Yeast Transformation II™ kit was used to prepare and transform yeast competent cells. Yeast plasmids were recovered by Zymoprep™ Yeast Plasmid Miniprep I kit and transformed into *E*. *coli* DH10B for propagation. PEG-mediated transformation was used to obtain transformants of *A*. *oryzae* as previously described (Lv et al. 2017).

### HPLC and LC–MS detection

To screen positive transformants, mycelia of single transformant grown on CD agar plate were resuspended in 300 μL methanol and the supernatant dealt with 0.22 μm filter was subjected to LC–MS detection. LC–MS analysis was performed on Shimadzu LCMS-2020 equipped with a Shim-pack GIST-HP C18 column (2.1 × 50 mm, 2 μm, Tokyo, Japan). Elution was subjected to a linear gradient [H_2_O containing 0.1% formic acid (A) and CH_3_CN (B); 0.2 mL·min^−1^; 10–100% B (0–10 min), 100% B (10–12 min), 100–10% B (12–12.01 min), 10% B (12.01–15 min); 360 nm]. For positive transformant AO-*rugABEFGH*-1 product analysis, the transformant was cultured in 100 mL MPY liquid medium for 5 days under continuous shaking at 220 rpm and extracted with 100 mL ethyl acetate. The organic phase was dried under reduced pressure and dissolved in 2 mL methanol for HPLC analysis. HPLC analysis was performed on an Agilent 1200 HPLC system with a DAD detector equipped with a Shim-pack GIS C18 column (250 × 4.6 mm, 5 μm, Tokyo, Japan). Elution was subjected to a linear gradient [H_2_O containing 0.1% formic acid (A) and MeOH (B); 1 mL·min^−1^; 10–100% B (0–15 min), 100% B (15–25 min), 100–10% B (25–26 min), 10% B (26–30 min); 360 nm].

## Results

### Construction of plug-and-play vectors

**Fig. 2:**
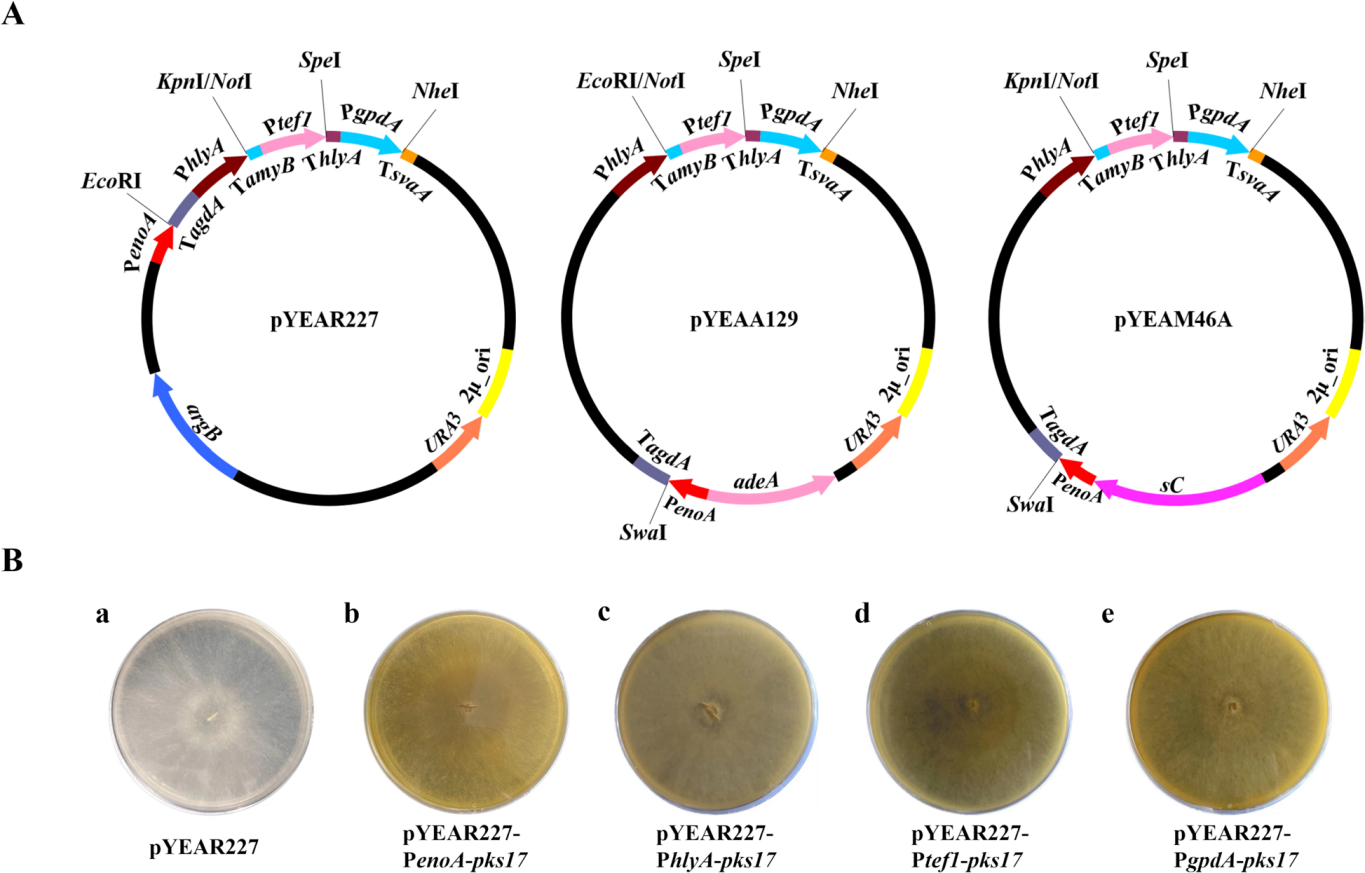
Verification of the selected constitutive promoters. **A** Maps of plug-and-play vectors pYEAR227, pYEAA129, and pYEAM46A. **B** Verification of the expression of **b** P*enoA*, **c** P*hlyA*, **d** P*tef1*, and **e** P*gpdA* in *A*. *oryzae* NSAR1 grown on CD agar plates. The transformant harboring empty pYEAR227 (**a**) was used as a control. *Pc21g16000* (*pks17*) encoding the biosynthesis of a yellow precursor was used as a reporter gene, and thus it can be judged whether the four promoters were functional in *A*. *oryzae* NSAR1 by plate color change

The six original vectors [pAdeA (Jin et al. 2004b), pTAex3 (Fujii et al. 1995), pUSA (Yamada et al. 2003), pUNA (Yamada et al. 1999), pBARI (Matsuda et al. 2014), pPTRI (Kubodera et al. 2000)] are widely employed in the reconstruction of natural product biosynthetic pathways. But each of these vectors bears at most one expression cassette (Fig. 1A). To express multiple genes in one vector, genes are usually ligated into vectors (pTAex3, pUNA, and pUSA) bearing expression cassettes to construct plasmids expressing single gene, and then expression structures of promoter-gene-terminator are amplified from those plasmids and introduced into the target vectors (Fig. 1B). The similar strategy was conducted in reconstructing the biosynthetic pathway of helvolic acid (Lv et al. 2017) and rugulosin A (Han et al. 2021). Although multiple gene expression vectors were developed, the same promoters were employed in one vector (Tagami et al. 2014), or the same restriction sites were inserted behind the promoters (Lazarus et al. 2014; Pahirulzaman et al. 2012; Yamane et al. 2017) (Fig. 1A). It might result in inconvenience in reconstructing biosynthetic pathways and inactivation of genes through undesirable crossovers in the host (Lazarus et al. 2014). Therefore, it is necessary to develop vectors that is more convenient for use. Since the expression of transformants on CD agar plates should be ensured so that LC–MS detection could be introduced for transformant screening, the commonly used inducible promoter P*amyB* was abandoned and four strong constitutive promoters, P*enoA*, P*hlyA*, P*tef1*, and P*gpdA*, were selected. In the meanwhile, four terminators, T*agdA*, T*amyB*, T*hlyA*, and T*svaA*, were matched and unique restriction sites were inserted between promoters and terminators. To improve the reconstruction efficiency of the natural product biosynthetic pathway, the amplification and screening cassette of yeast was introduced into the vectors for multi-fragment homologous recombination in yeast. Combining above functional elements, we developed pYEAR227 based on pTAex3 (Fig. 2A). Notably, although the amplification and screening cassette of yeast was inserted into the vectors, the methods of reconstructing biosynthetic pathways are not restricted within homologous recombination in yeast, and cloning kits can also be selected according to preference. It was reported that P*melO* originated from *A*. *oryzae* was little expressed in *A*. *oryzae* M-2-3 (Ishida et al. 2004). To avoid the same problem being encountered, *Pc21g16000* (*pks17*) encoding the biosynthesis of a yellow precursor was placed under the control of the four promoters, and the resultant plasmids were transformed into *A*. *oryzae*. Therefore, it can be judged whether the four promoters were functional in *A*. *oryzae* NSAR1 by observing the color change of the plates. Fortunately, yellow pigments were produced (Fig. 2B), indicating that the four promoters were functional in the host. Inspired by the successful construction of pYEAR227, we further developed pYEAA129 and pYEAM46A (Fig. 2A) based on pAdeA and pUSA. With the three multiple gene expression vectors developed here, a dozen genes could be transformed into the host in a round of transformation and the majority of fungal BGCs could be expressed in *A*. *oryzae*. Because the expression cassettes comprising promoters and terminators have been introduced into the plug-and-play vectors, genes amplified from genomic DNA can be inserted into the desired vectors directly. Thus, the construction of single gene expression plasmids for amplifying promoter-gene-terminator expression structures is avoided and 3 days can be saved (Fig. 1B).

### LC–MS detection to screen positive transformants

**Fig. 3:**
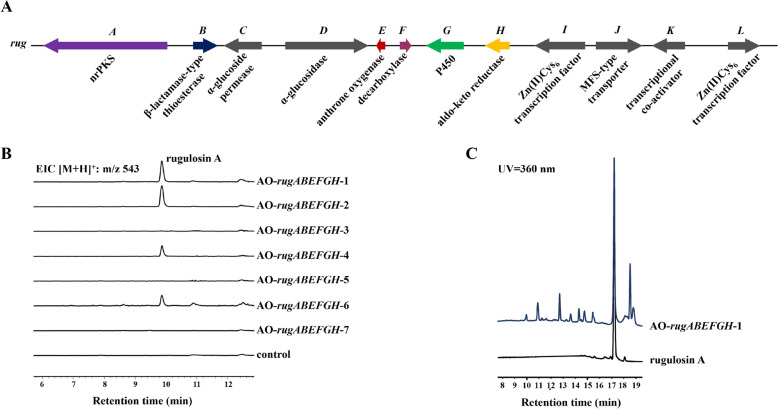
Validation of the strategy of employing plug-and-play vectors and LC–MS screening. **A** The organization of *rug* BGC. The genes that do not incorporate in rugulosin A production were in *gray*. **B** LC–MS detection of rugulosin A produced by *rug* BGC transformants. Mycelia of transformants grown on CD agar plates were resuspended in 300 μL methanol and the supernatant dealt with 0.22 μm filter was subjected to LC–MS detection. The extracted ion chromatogram (EIC) was extracted at m/z 543 [M+H]^+^ for rugulosin A. **C** HPLC profiles of metabolites produced by positive transformant AO-*rugABEFGH*-1. The transformant was cultivated in MPY liquid medium for 5 days and the extract was subjected to HPLC analysis

A common challenge in using *A*. *oryzae* to express natural products is the screening of positive transformants. Random integration of transformed plasmids leads to variable gene expression levels (Pahirulzaman et al. 2012), necessitating labor-intensive PCR and fermentation-based re-screening. CRISPR-Cas9-guided homologous recombination was successfully applied in *A*. *oryzae* NSPlD1 (Maruyama and Kitamoto 2008) for natural product expression, and it greatly increased the positive transformant rate (Katayama et al. 2016; Liu et al. 2019; Nagamine et al. 2019). However, the knock-in rate ranged from 80 to 90% (Nagamine et al. 2019). Therefore, the process of screening positive transformants might also be required, and an efficient method for screening positive transformants is still necessary. Furthermore, given that *A*. *oryzae* NSAR1 (Jin et al. 2004a), in which the transformed plasmids are randomly integrated into the genome, is the most widely used heterologous expression strain of *A*. *oryzae* (Mózsik et al. 2022), it is still significant to establish a robust means of screening positive transformants. LC–MS with sensitivity, speed, and throughput has been common in laboratories for high-throughput qualitative and quantitative analysis, and it has been used for metabolite analysis of the extracts of *A*. *oryzae* mycelia grown in/on rich medium (Jiang et al. 2022; Pahirulzaman et al. 2012). However, to the best of our knowledge, LC–MS has not been reported to be used directly to screen positive transformants grown on CD agar plates. Previously optimized vectors (Fujii et al. 1995; Lazarus et al. 2014; Pahirulzaman et al. 2012; Tagami et al. 2014; Yamada et al. 1999, 2003; Yamane et al. 2017) favored the use of inducible promoter P*amyB* that is repressed on CD agar plates with sucrose or glucose as sole carbon source. It may result in the lack of a direct method for screening positive transformants on CD agar plates. Since pYEAA129, pYEAR227, and pYEAM46A bears four strong constitutive promoters and genes are expressed on CD agar plates, we attempted to introduce LC–MS detection to screen positive transformants to simplify the screening process. To verify our strategy, the *rug* BGC (Fig. 3A) containing 6 genes was reconstructed using pYEAR227 and pYEAM46A. The BGC was transformed into *A*. *oryzae* in a round of transformation and 7 transformants were obtained. Then mycelia of single transformant grown on CD agar plate were resuspended in 300 μL methanol and the supernatant dealt with 0.22 μm filter was subjected to LC–MS detection. The results of LC–MS detection were shown in Fig. 3B. Among the 7 transformants, 4 transformants expressed the final product rugulosin A. It seemed that transformants AO-*rugABEFGH*-1 and AO-*rugABEFGH*-2 were more suitable for fermentation to isolate products (Fig. 3B, Fig. S1). AO-*rugABEFGH*-1 was selected for further verification in rich medium. After 5 days of fermentation in MPY medium, the production of rugulosin A was also detected by HPLC (Fig. 3C). Thus, the successful expression and detection of rugulosin A in the transformants indicates that the multiple gene expression vectors we developed perform well in expressing natural products and the strategy of employing LC–MS detection to screen positive transformants is feasible. Furthermore, employing LC–MS detection to screen positive transformants, we can save about 7 days.

### Verification of inducible promoters for deleterious gene expression

**Fig. 4:**
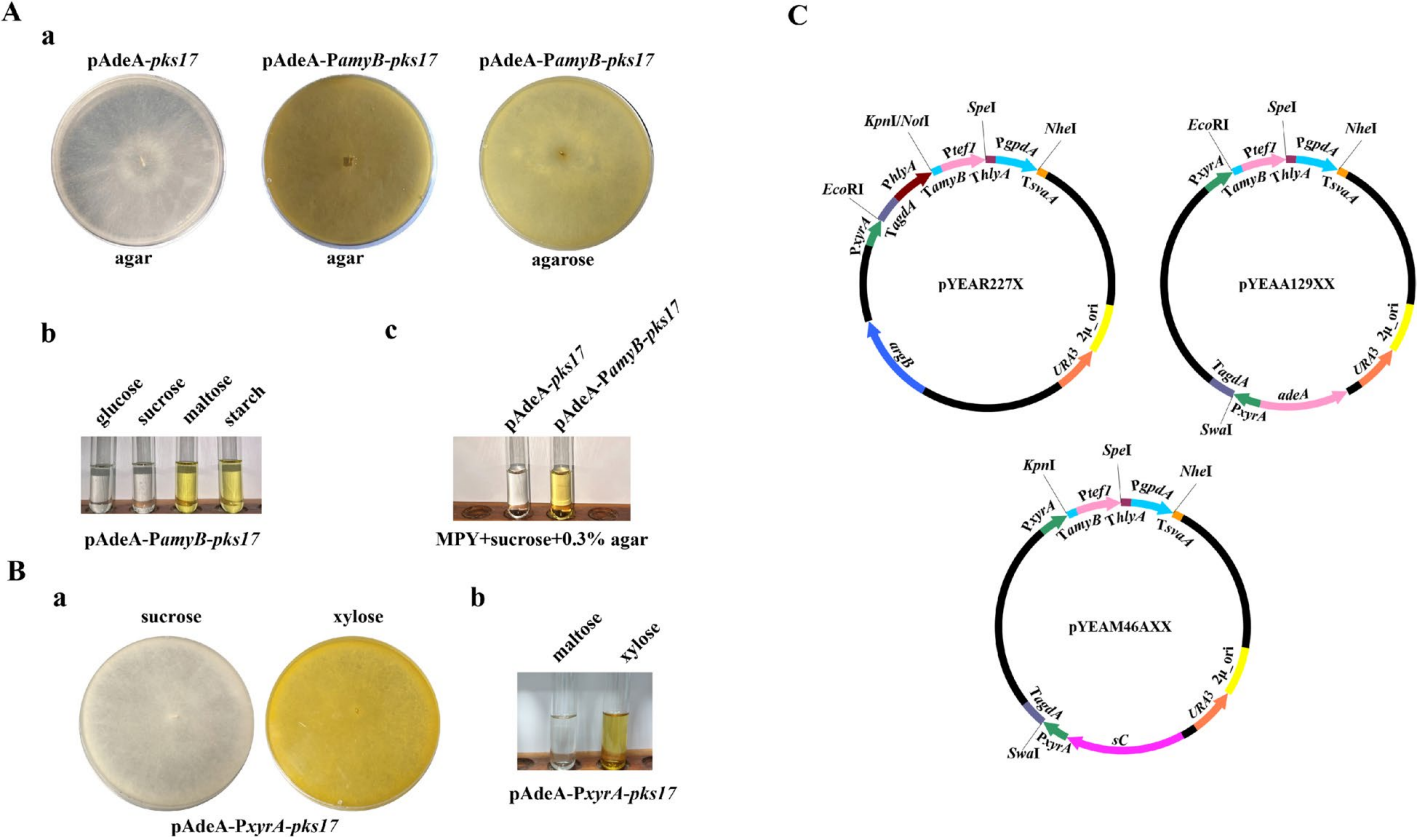
Selection and verification of inducible promoters P*amyB* and P*xyrA*. **A** Verification of the inducible promoter P*amyB*. **a** Effects of coagulants on P*amyB* induction. AO-P*amyB*-*pks17* was cultivated on CD plates with agar (*middle*), agarose (*right*) as coagulants. The transformant harboring pAdeA-*pks17* in which no promoter was placed upstream of *pks17* was used as a control (*left*). These transformants were cultivated on CD plates with sucrose as sole carbon source. **b** Effects of carbon sources on P*amyB* induction. AO-P*amyB*-*pks17* was cultivated in MPY liquid medium with glucose, sucrose, maltose, and starch (form *left* to *right*) as sole carbon source for 5 days, and then the culture was extracted with equal volume of ethyl acetate. 3 mL ethyl acetate extract was transferred into tubes for data collection. **c** Transformant harboring pAdeA-*pks17* (*left*) and transformant AO-P*amyB*-*pks17* (*right*) were cultivated in MPY liquid medium supplemented with 0.3% agar. Sucrose was as sole carbon source. **B** Verification of the inducible promoter P*xyrA*. **a** AO-P*xyrA*-*pks17* was cultivated on CD agar plates with sucrose (*left*) and xylose (*right*) as sole carbon source. **b** AO-P*xyrA*-*pks17* was cultivated in MPY liquid medium with maltose (*left*) and xylose (*right*) as sole carbon source. Data collection was performed as above. **C** Maps of P*xyrA*-contained vectors pYEAR227X, pYEAA129XX, and pYEAM46AXX

While constitutive promoters simplify the screening of positive transformant, their use is unsuitable for deleterious genes that hinder host biomass accumulation. Hence, we attempted to screen some inducible promoters to express deleterious genes. Maltose- and starch-induced P*amyB* is frequently employed to regulate gene expression, but it is not tightly regulated (Tsuchiya et al. 1992). To verify whether P*amyB* was feasible to express deleterious genes, *pks17* was employed as reporter gene and placed under the control of P*amyB*. CD agar plates and MPY liquid medium, usually used for transformant subculture and fermentation, were utilized to cultivate the transformants harboring P*amyB* controlled *pks17* (termed as AO-P*amyB*-*pks17*). Unfortunately, AO-P*amyB*-*pks17* produced yellow pigments on CD agar plates with sucrose as carbon source (Fig. 4A), which indicated that P*amyB* failed in regulating gene expression. However, when AO-P*amyB*-*pks17* was cultured in MPY liquid medium with glucose or sucrose as sole carbon source, yellow pigments were not produced. In the meanwhile, yellow pigments were produced again when maltose or starch was as sole carbon source (Fig. 4A). It meant that P*amyB* regulated gene expression well in liquid medium and there might be inducers unknown in agar plates. It was speculated that agar induced P*amyB* expression. To verify our view, MPY medium with sucrose as sole carbon source was supplemented with 0.3% agar and the modified medium was used to cultivate AO-P*amyB*-*pks17*. Unsurprisingly, yellow pigments were produced (Fig. 4A). It indicated that agar was also an inducer of P*amyB*. To repress the expression of P*amyB* on CD agar plates, agar was replaced with agarose. In the early stages of culture, yellow pigments were not observed; once the hyphae filled the plate, yellow pigments were produced again although the expression strength was reduced (Fig. 4A). Physical barriers to hyphal extension enhance *glaB* expression in solid-state culture (Ishida et al. 2000). It seemed that physical barriers to hyphal extension also affected P*amyB* expression and P*amyB* is not an ideal inducible promoter although it is wildly employed to regulate gene expression.

To screen an ideal inducible promoter for deleterious gene expression, we turned to other reported inducible promoters. P*glaB* is used to conditionally express genes, but it is not tightly regulated either (Ishida et al. 1998). P*sorA* and P*sorB* induced by sorbitol are strictly controllable but much weaker (Jeennor et al. 2022; Oda et al. 2016). P*thiA* is tightly regulated but not functional in alkaline conditions (Shoji et al. 2005). A novel xylose-induced promoter P*xyrA* was thought to be regulated tightly and expressed efficiently (Jeennor et al. 2022). To verify whether P*xyrA* performed as described, *pks17* was placed under the control of P*xyrA* and transformed into *A*. *oryzae*. The yellow pigments produced by the transformant harboring P*xyrA* controlled *pks17* (termed as AO-P*xyrA*-*pks17*) were observed when sucrose was replaced by xylose (Fig. 4B). AO-P*xyrA*-*pks17* was then inoculated into MPY liquid medium and yellow pigments were produced only when xylose was added (Fig. 4B). It indicated that P*xyrA* regulated gene expression tightly in tested conditions and it was more suitable to be employed to regulate gene expression than P*amyB*. With this tightly regulated promoter in hand, we inserted it into pYEAR227, pYEAA129, and pYEAM46A to develop pYEAR227X, pYEAA129XX, and pYEAM46AXX (Fig. 4C) for deleterious gene expression.

## Discussion

The heterologous expression of biosynthetic gene clusters in *A*. *oryzae* typically involves three critical steps: reconstruction of the natural product biosynthetic pathway, transformation of expression vectors into the host, and screening of positive transformants. There are several speed bumps in these three parts. In this study, we developed multiple gene expression vectors to reconstruct BGCs and introduced LC–MS detection to screen positive transformants. However, the methods of increasing the transformation efficiency and the ratio of positive transformants was not mentioned. Liu et al. significantly increased the ratio of positive transformants through CRISPR/Cas9-mediated targeted integration, but it did not reach 100% (Liu et al. 2019). We have tried to introduce AMA1 into vectors so that the transformation efficiency can be greatly improved and the proportion of positive transformants can reach 100% in theory because of vectors extrachromosomal maintenance (Aleksenko and Clutterbuck 1997). However, the yield of those transformants was extremely low, making it difficult to obtain enough products for analysis (data not shown). Therefore, it is more practicable to increase the ratio of positive transformants through CRISPR/Cas9-mediated targeted integration. With appropriate modification, the strategy described in this paper can be applied together with targeted integration, and it will facilitate the discovery of natural products using *A*. *oryzae*.

Due to unidentified components of agar and physical barriers to hyphal extension might induce P*amyB* expression, a tightly regulated promoter P*xyrA* was selected to modulate deleterious gene expression. However, it is concerned whether xylose-induced P*xyrA* could collaborate well with promoters originated from glycolytic pathway such as P*gpdA* and P*enoA* to express natural products. It was reported that *enoA* transcript levels depend on the carbon source (Inoue et al. 2020) but the expression of P*gpdA* originated from *A*. *nidulans* is not significantly interfered with xylose (Baldin et al. 2022). Further exploration should be conducted when P*xyrA* and constitutive promoters are employed simultaneously. Fortunately, the vectors, especially the P*xyrA*-contained vectors, are modular, and thus expression cassettes could be substituted rapidly and conveniently on demand.

## Conclusions

**Fig. 5:**
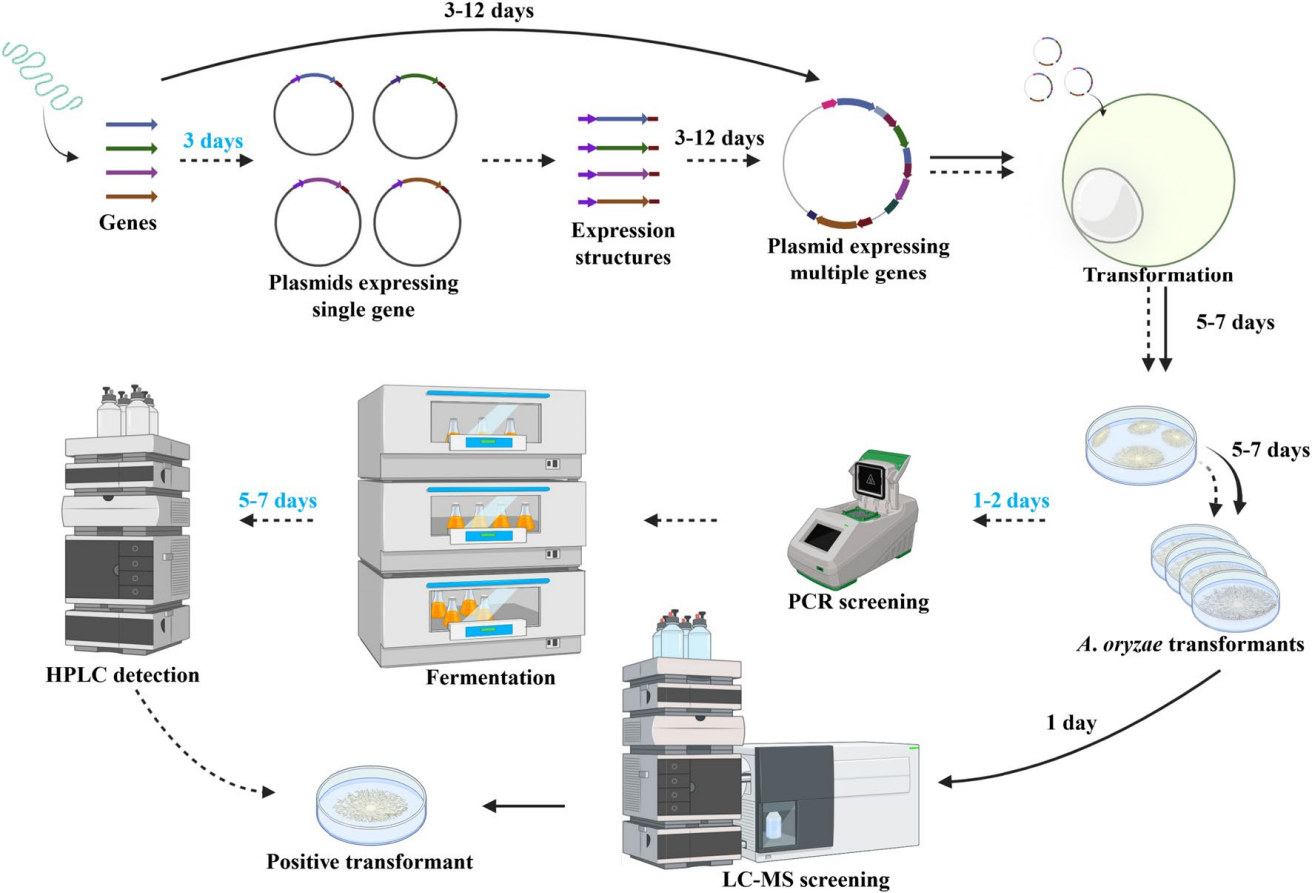
The scheme of reconstructing the natural product biosynthetic pathway and screening positive transformants. The workflow of employing the strategy presented in this paper to reconstruct the biosynthetic pathway and screen positive transformants was represented by *solid arrows*. The workflow of employing the six original vectors (pAdeA, pTAex3, pUSA, pUNA, pBARI, pPTRI) and PCR plus fermentation to reconstruct the biosynthetic pathway and screen positive transformants was represented by *dashed arrows*. The time that could be saved was highlighted in *blue*

*A*. *oryzae* is widely used to express fungal biosynthetic gene clusters, but the vectors are not convenient for use and the process of screening positive transformants is time- and effort-consuming. Herein, three vectors termed as pYEAA129, pYEAR227, and pYEAM46A are developed, in which four strong constitutive promoters were inserted and unique restriction sites were deployed between promoters and terminators. To simplify the process of screening positive transformants, LC–MS is introduced. With the tools and strategy presented here, approximately 10 days can be saved when expressing fungal biosynthetic gene clusters in *A*. *oryzae*, and many efforts can be saved (Fig. 5).

## Supplementary Information

Additional file 1.

## Data Availability

Not applicable.
