# Supplementary material for: Strategy of employing plug-and-play vectors and LC–MS screening to facilitate the discovery of natural products using Aspergillus oryzae
Source: Bioresour Bioprocess. 2025 Jan 8;12(1):2. doi: 10.1186/s40643-024-00833-w (PMC11711427; doi:10.1186/s40643-024-00833-w)
Supplement: Supplementary file 1 — Additional file 1. [file 40643_2024_833_MOESM1_ESM.docx]

Table S1 Primers used in this study

| Primer | Sequence (5´-3´) | Purpose |
| --- | --- | --- |
| pAdeA-d5-fw | ctggacatgacggaatcggatccccgggtaccgagctcCTACCCCACCATCCCACTATT | amplification of P*enoA* for construction of pYEAA129, pYEAM46A |
| T*agdA*-*Swa*1-P*enoA*-rv | gtctaggctatcctgttacgcttccttca**ATTTAAAT**TTTGACGAGCTGCGGAATTGGT |  |
| P*enoA*-*Swa*1-T*agdA*-fw | actgaccaattccgcagctcgtcaaa**ATTTAAAT**TGAAGGAAGCGTAACAGGATAGCCT | amplification of T*agdA* for construction of pYEAA129, pYEAM46A |
| pAdeA-T*agdA*-rv | gttttcccagtcacgacgttgtaaaacgacggccagtGGCAGTAACCCATTCCCGGTTC |  |
| pAdeA-P*hlyA*-fw | tctaatacgactcactatagggaaagcttgcatgcTACAGCATGGTCTGGATTCCAATC | amplification of P*hlyA* for construction of pYEAA129 |
| T*amyB*-*Not*1-*Eco*R1-P*hlyA*-rv | ccttcacgagctactacagatc**GCGGCCGCGAATTC**GGTGTTGTGGTGTGAAGGGTGAT |  |
| P*hlyA*-*Eco*R1-*Not*1-T*amyB*-fw | cacccttcacaccacaacacc**GAATTCGCGGCCGC**GATCTGTAGTAGCTCGTGAAGGGT | amplification of T*amyB* for construction of pYEAA129, pYEAR227, and pYEAM46A |
| P*tef1*-T*amyB*-rv | tgcccctcatttagatcaatcccttactgtctcggaccttCTTTCCTATAATAGACTAGCGTGCTTG |  |
| P*tef1*-fw | AAGGTCCGAGACAGTAAGGGATTGAT | amplification of P*tef1* for construction of pYEAA129, pYEAR227, and pYEAM46A |
| *Spe*1-P*tef1*-rv | AGA**ACTAGT**TTTGAAGGTGGTGCGAACTTTGTAGT |  |
| P*tef1*-*Spe*1-T*hlyA*-fw | ACCACCTTCAAA**ACTAGT**TCTCTCTCCCCTATACGTGATACCGT | amplification of T*hlyA* for construction of pYEAA129, pYEAR227, and pYEAM46A |
| P*gpdA*-T*hlyA*-rv | tcatctctttggaatttttttaccattgtttattcttttGATGCAAATTGGAGTTAAATATTAACTAAC |  |
| P*gpdA*-fw | AAAAGAATAAACAATGGTAAAAAAATTCCAAAGAG | amplification of P*gpdA* for construction of pYEAA129, pYEAR227, and pYEAM46A |
| *Nhe*1-P*gpdA*-rv | GAT**GCTAGC**TGTTTAGATGTGTCTGTTGGTGT |  |
| P*gpdA*-*Nhe*1-T*svaA*-fw | ACATCTAAACA**GCTAGC**ATCATTTTCCCGCTTTGATCTG | amplification of T*svaA* for construction of pYEAA129, pYEAR227, and pTEAM46A |
| Ura2uori-T*svaA*-rv | caaggcgattaagttgggtaATCAAGCATAACTACAACAGGGCAAG |  |
| T*svaA*-ura2uori-fw | ctgttgtagttatgcttgatTACCCAACTTAATCGCCTTGCAG | amplification of *URA3* and 2μ_ori for construction of pYEAA129, pYEAR227, and pYEAM46A |
| pAdeA-ura2uori-rv | cctgtggactcgtatgctgttgcgcagaatccatatgaGCGTATCACGAGGCCCTTTCG |  |
| pTAex3-*Xho*l-d5-fw | cggagtggctcacgcgcccgccaccacacccgaag**CTCGAG**CTACCCCACCATCCCACTATT | amplification of P*enoA* for construction of pYEAR227 |
| T*agdA*-*Eco*R1-P*enoA*-rv | gggtctaggctatcctgttacgcttccttca**GAATTC**TTTGACGAGCTGCGGAATTGGT |  |
| P*enoA*-*Eco*R1-T*agdA*-fw | cgactgaccaattccgcagctcgtcaaa**GAATTC**TGAAGGAAGCGTAACAGGATAGCCT | amplification of T*agdA* for construction of pYEAR227 |
| P*hlyA*-T*agdA*-rv | tatctgctgcgtggattggaatccagaccatgctgtaGGCAGTAACCCATTCCCGGTTC |  |
| T*agdA*-P*hlyA*-fw | tattacagctagagaaccgggaatgggttactgccTACAGCATGGTCTGGATTCCAATC | amplification of P*hlyA* for construction of pYEAR227, pYEAM46A |
| T*amyB*-*Not*1-*Kpn*1-P*hlyA*-rv | ccttcacgagctactacagatc**GCGGCCGCGGTACC**GGTGTTGTGGTGTGAAGGGTGAT |  |
| P*hlyA*-*Kpn*1-*Not*1-T*amyB*-fw | cacccttcacaccacaacacc**GGTACCGCGGCCGC**GATCTGTAGTAGCTCGTGAAGGGT | amplification of T*amyB* for construction of pYEAR227, pYEAM46A |
| pTAex3-ura2uori-rv | gaagtaccatacagtaccgcgttatgaaaacacattaatccGCGTATCACGAGGCCCTTTCG | amplification of *URA3* and 2μ_ori for construction of pYEAR227 |
| pUSA-d5-fw | acttcctgtcatacgggcagctattgccaagagaagctCTACCCCACCATCCCACTATT | amplification of P*enoA* for construction of pYEAM46A |
| pUSA-T*agdA*-rv | acgttgtaaaacgacggccagtgccaagcttgcatgcGGCAGTAACCCATTCCCGGTTC | amplification of T*agdA* for construction of pYEAM46A |
| pUSA-P*hlyA*-fw2 | cggataacaatttcacacaggaaacagctatgaccTACAGCATGGTCTGGATTCCAATC | amplification of P*hlyA* for construction of pYEAM46A |
| pUSA-ura2uori-rv | atttggatttttatatccaagatcgactctagaggatcGCGTATCACGAGGCCCTTTCG | amplification of *URA3* and 2μ_ori for construction of pYEAM46A |
| P*tef1*-PC-fw | ttacaaagaactacaaagttcgcaccaccttcaaaATGGAAGGCCCCGGTCAT | amplification of *pks17* for construction of pYEAR227-P*tef1*-*pks17* |
| T*hlyA*-PC-rv | taaaagactacggtatcacgtataggggagagagaTTACACAGAAGCCATAGAGTTAG |  |
| P*gpdA*-PC-fw | agctaccccgccacaccaacagacacatctaaacaATGGAAGGCCCCGGTCAT | amplification of *pks17* for construction of pYEAR227- P*gpdA* -*pks17* |
| T*svaA*-PC-rv | actgggaaccgaccagatcaaagcgggaaaatgatTTACACAGAAGCCATAGAGTTAG |  |
| P*hlyA*-PC-fw | ggtctcacatcaatcacccttcacaccacaacaccATGGAAGGCCCCGGTCAT | amplification of *pks17* for construction of pYEAR227- P*hlyA* -*pks17* |
| T*amyB*-PC-rv | atatactctccacccttcacgagctactacagatcTTACACAGAAGCCATAGAGTTAG |  |
| P*enoA*-PC-fw | agttagtcgactgaccaattccgcagctcgtcaaaATGGAAGGCCCCGGTCAT | amplification of *pks17* for construction of pYEAR227- P*enoA* -*pks17* |
| T*agdA*-PC-rv | atgtgggtctaggctatcctgttacgcttccttcaTTACACAGAAGCCATAGAGTTAG |  |
| P*tef1*-rugA-fw | ctacaaagttcgcaccaccttcaaaATGGAACTCGTGTATTTCAG | amplification of *rugA* for construction of pYEAR227-*rugA* |
| T*hlyA*-*rugA*-rv | ggtatcacgtataggggagagagaTTAACTATAATATTCCTCCAACCAC |  |
| P*gpdA*-*rugB*-fw | ccacaccaacagacacatctaaacaATGGGGGACGACAAAGG | amplification of *rugB* for construction of pYEAR227-*rugAB* |
| T*svaA*-*rugB*-rv | gaccagatcaaagcgggaaaatgatTCATGTCGGAATTTCCTGAAC |  |
| P*hlyA*-*rugE*-fw | caatcacccttcacaccacaacaccATGACGAGCCTTTCAGTC | amplification of *rugE* for construction of pYEAM46A-*rugEFGH* |
| T*amyB*-*rugE*-rv | cacccttcacgagctactacagatcCTATATACCGACCTCTTTGAGAAT |  |
| P*gpdA*-*rugF*-fw | ccacaccaacagacacatctaaacaATGGCCACCGTCGACAAG | amplification of *rugF* for construction of pYEAM46A-*rugEFGH* |
| T*svaA*-*rugF*-rv | gaccagatcaaagcgggaaaatgatTTAACACTCCTTGAAGCCGTCAAC |  |
| P*enoA*-*rugG*-fw | ctgaccaattccgcagctcgtcaaaATGGACCTGAACTCGCAGG | amplification of *rugG* for construction of pYEAM46A-*rugEFGH* |
| T*agdA*-*rugG*-rv | ggctatcctgttacgcttccttcaTTAAGCCTTCTCCCTAGGAACATAC |  |
| P*tef1*-*rugH*-fw | ctacaaagttcgcaccaccttcaaaATGTCCGCCACAATTGAAAAC | amplification of *rugH* for construction of pYEAM46A-*rugEFGH* |
| T*hlyA*-*rugH*-rv | cggtatcacgtataggggagagagaTCACTGAAAGTATGGTCCACAAAC |  |
| pAdeA-*Swa*1-PC-fw | ctcactatagggaaagcttgcatgc**ATTTAAAT**ATGGAAGGCCCCGGTCAT | amplification of *pks17*-T*hlyA* for construction of pAdeA-*pks17* |
| pAdeA-T*hlyA*-rv | gactagtagatcctctagagtcgacGATGCAAATTGGAGTTAAATATTAACTAACAAAT |  |
| pAdeA-PamyB616-fw | ctcactatagggaaagcttgcatgcAATTCATGGTGTTTTGATCATTTT | amplification of P*amyB* for construction of pAdeA-P*amyB*-*pks17* |
| PC-PamyB616-rv | gatatacatgaccggggccttccatAAATGCCTTCTGTGGGGTTT |  |
| pAdeA-P*xyrA*-fw | ctcactatagggaaagcttgcatgcCTGGCATAGGAGTTTACGGGGAGGGAT | amplification of P*xyrA* for construction of pAdeA-P*xyrA*-*pks17* |
| PC-P*xyrA*-rv | gatatacatgaccggggccttccatTGTGATTATTGTGTGTAGAGATTGCGCTT |  |
| 1B-1A-fw | CTTGCATGC**GAATTC**GATCTGTAGTAGCTCGTGAAGGGT | construction of pYEAA129XX and pYEAM46AXX |
| 1B-1A-rv | GCTTCCTTCA**ATTTAAAT**GCGTATCACGAGGCCCTTTC |  |
| 1A-1B-fw | GATACGC**ATTTAAAT**TGAAGGAAGCGTAACAGGATAGC |  |
| 1A-1B-rv | ACTACAGATC**GAATTC**GCATGCAAGCTTTCCCTATAGTG |  |
| 1-*Xho*1-P*xyrA*-fw | aagacgaaagggcctcgtgatacgc**CTCGAG**CTGGCATAGGAGTTTACGGGGAGGGAT |  |
| T*agdA*-*Swa*1-P*xyrA*-rv | ctatcctgttacgcttccttca**ATTTAAAT**TGTGATTATTGTGTGTAGAGATTGCGCTT |  |
| 1-*Not*1-P*xyrA*-fw | cactatagggaaagcttgcatgc**GCGGCCGC**CTGGCATAGGAGTTTACGGGGAGGGAT |  |
| T*amyB*-*Kpn*1-P*xyrA*-rv | acccttcacgagctactacagatc**GGTACC**TGTGATTATTGTGTGTAGAGATTGCGCTT |  |
| T*amyB*-*Eco*R1-P*xyrA*-rv | acccttcacgagctactacagatc**GAATTC**TGTGATTATTGTGTGTAGAGATTGCGCTT |  |
| PxyrAadeA-rv | atccctccccgtaaactcctatgccagGAGCTCGGTACCCGGGGAT |  |
| AadeA-fw | TGAAGACGAAAGGGCCTCGTGATAC |  |
| PxyrAsC-rv | cctccccgtaaactcctatgccagAGCTTCTCTTGGCAATAGCTG |  |
| 2-P*xyrA*-fw | cacgcgcccgccaccacacccgaag**CTCGAG**CTGGCATAGGAGTTTACGGGGAGGGAT | construction of pYEAR227X |
| T*agdA*-*Eco*R1-rv | ggctatcctgttacgcttccttca**GAATTC**TGTGATTATTGTGTGTAGAGATTGCGCTT |  |

Table S2 The sequences of the promoters, terminators, and *pks17*

| Fragment | Sequence (5´-3´) |
| --- | --- |
| P*hlyA* | TACAGCATGGTCTGGATTCCAATCCACGCAGCAGATATCTCTTTTACGCAGTAACTATTCGATAGCCATTTCCGTTCAGATCAGCCGTCGGATCCGAGGAGCGACGACATCAATGCGTGTTATTAGTCAAATTTTGGAGGGGTTGCGTGCCCACTGCAGGCAGATGTAGCCGTGGCACCACAACACCGGCCAGCCCTGGATTGGGTGGTGGAACCAAGATATGAGAACCAGTATCTATTGGCACGGAGGCGTTTCCGGAGCCTGCCGCGTGTGATACGTGCAGAGTCAATTCCTACGGACTGTCTGGGGTAGGATCACAACTAATCAGTTGCAGCGATGGTTTGACAACAGGGGTCGATCAAAGTTTCCGAAGAATAGGAAGCGAGGACAGCACCAAGGCCGCTGAACCACAGGAAACAAAGGAAGGAAAAACACAACAAAACCAAAGACAGACACATAGGAAATGACATACTTACCAGAGATAAGATGAAAAGCACCATGGGGAGGGAGGGTATGGATGGGGAAGTTGATCAACAGTCTGAAACCCGCCCGAAATGAATGCATGACGCGACGATTCCATCTCCACCTAAGCTTCATCCCGTCAACCTCTAACAACGCGCTCGGAGGAGAAAAAGAGGGGGATCAACAGCAATCTAGGTTGTTGTTCCCCCAGGATTTCTCCGCAAAGAATGTTTAGGGTTCCGTGGATCGGGTGACCGAATCGGCCAACCGCATTGTCTGATCGTCTCGTCATATGATCCAGTGCATGACGTCTTCCATCAATCCATCACCCCAGACTTGAATCCTCACATTAATTCAACAATTGTCGCTCGGGGAAATCAATAAATACCCGCTCGTCTCCTCCCTCCCTCGTGCTGGTTCAGTTGATTGTTCACTCATCGACTTTATCAATCTTCCATTGACCATTCCAGGCTTGTCGCCCACTCATATAATCTTCTTTCCCCGGTCTCACATCAATCACCCTTCACACCACAACACC |
| T*amyB* | GATCTGTAGTAGCTCGTGAAGGGTGGAGAGTATATGATGGTACTGCTATTCAATCTGGCATTGGACAGTGAGTTTGAGTTTGATGTACAGTTGGAGTCGTTACTGCTGTCATCCCCTTATACTCTTCGATTGTTTTTCGAACCCTAATGCCAAGCACGCTAGTCTATTATAGGAAAG |
| P*tef1* | AAGGTCCGAGACAGTAAGGGATTGATCTAAATGAGGGGCACACAGCAGCGATCATATGTGAAAGAAAAACTAGAAAGGCAAAAAAAGAAAAGAAAAAGAGAAGAAAATTTTTCCTAGGTATCCGTAGTCTGTCAACATTGCCTCTTTGCCTCCTAACAGCATATGTACGGAGTATCTGTACGTACTCTAGATAGCGAGAGTAAAAAAAAAAAAGATTTTCACTGTGGACCAGACAGGCGCCACTCGGCCGGGCCACAGCTGCTTGGGTGTTGACCGGGAGCGGACCAATTAAGGACTCGAACGACCGCGGGGTTCAAATGCAAACAAGTACAACACGCAGCAAACGAAGCAGCCCACCACTGCGTTGATGCCCAGTTTGACTGTCCGAAATCCACCGGAAAGGTGGAAACATACTATGTAACAATCAGAGGGAAGAAAAAATTTTTATCGACGAGGCAGGATAGTGACTGATGGTGGGGTCATGGTCGGGTCTCCGAGCGAAAGAGAACCAAGGAAACAAGATCAACGAGGTTGGTGTACCCAAAAGGCCGCAGCAACAAGAGTCATCGCCCAAAAGTCAACAGTCTGGAAGAGACTCCGCCGTGCAGATTCTGCGTCGGTCCCGCACATGCGTGGTGGGGGCATTACCCCTCCATGTCCAATGATAAGGGCGGCGGTCGAGGGCTTAAGCCCGCCCACTAATTCGCCTTCTCGCTTGCCCCTCCATATAAGGATTCCCCCTCCTTCCCCTCCCACAACTTTTTTCCTTCTTTCTCTCTTCGTCCGCATCAGTACGTATATCTTTCCCCCATACCTCCTTTCCTACTCTTCTTCCATTCATTCAACTCTTCTCCTTACTGACATCTGTTTTGCTCAGTACCTCTACGCGATCAGCCGTAGTATCTGAGCAAGCTTTTCTACAGAATCTTTCTAGTATCTTACAAAGAACTACAAAGTTCGCACCACCTTCAAA |
| T*hlyA* | TCTCTCTCCCCTATACGTGATACCGTAGTCTTTTACGGGTGTAGTCCTTTTTTTCTTTTCATTATGAGTTCAACTTTTTTTTTTTTTTTTTTTTTTTTTTTTTTGTCGTCTCCATGTTCACGCGAGATGTTACATATTTTGATATCATACCAGAGAAACCCACTATATTAGGAACGAAGCTCGATTGATCAACCAATTAACAATCCCAAGTAATGAAGACGCCAATAGTGTAGCTGAATCAGCGTAGCAGGAACTGGTCACCAATGATTTGTTAGTTAATATTTAACTCCAATTTGCATC |
| P*gpdA* | AAAAGAATAAACAATGGTAAAAAAATTCCAAAGAGATGATTAATTGAAATTAAAAGAGAAAATAAAACCATGACAAGCGCGTGATCCTCATTTGCCTACCGGTGAATCTTTTCAGGAATCAGGAGAGCAGAAGAGGCTTAGAGAGATCGCTAAGCCCATGATATGAGTGTCGAGAGGGAAGAGGGGCAGTGGACCATAGTTGATCCGGTCCGATATCTCGGCCCGGAAACGGAAAGGTCACACCGAGTGCCCCTCATTTTTCCATTGCTTCCATCCATTAAGCTTGGGTGGGATGCTGTGGTCTGTAGTGTTAGTCTGTATGGCCAGATTGTAATTACATCATGCCCCTCTATGGGGATGCCTCAGGTATGGGACCCCAGGGTATCATTTCCCCCTCAATTGCTTGAACTACGGAACAAAGGACAAAAAGATAGAGTAATAGCCGGGATCGTCTTCCTCGTAGCCTAGGTAGTACTGCCCCCTCGATTCCGAAAAACTGGCAAAAGATTCACGAGATGGTAGGATTGAGTACCCGGCATGCTGGATTTGAGGCACGCTCATTGGCCAGACCGGTAGCTGCCGAGGAGAGGCAGAGTCCCAAATATCGTGAGTCTCCTGCTTTGCCCGGTGTATGAAACCGGAAAGGGCTGCTGGGAGCTGGGGAGCGGCGCAAGCCGGGAAAACAGCTGACAAGGACCCATTTCACTCTGGATCTTGAGGAGAGCTGTAGCTTTTGCCCCGTCTGTCCACCCGGTGACTGGATTAGTGACCTGGTCGTTGCGTCAGTCAACATTGCTCTTTTTTTATCTCCCCCTCCCCCGCCGTCCGACTTTTCTCCCCTTTTCTACTCTCTTCGTATACTCACCACTGCAATCACCTTATCCCTTTGTCTTCTTACTTAAAGTGAGTCGTCTCCCGCCCATCATTCCCTTTGGATCTCATAAAGCAGAGCCACTTTCAAGTGCCTACCGTTTCCCTTTCCACACAGATTGACTGACAGCTACCCCGCCACACCAACAGACACATCTAAACA |
| T*svaA* | ATCATTTTCCCGCTTTGATCTGGTCGGTTCCCAGTTAAGGTTAGTCTATCCGCAAATCCTTTGGTTTTGTCAGGGGTATTGACGTCACAGTCATCTATCCGAACGATCAATCACGACTGAATGTTCTATGTCTCATGGACGCGGTGGGTGGAAGTGGCTTGTTTTTTTTGCAACGTTTTCAATTTGTATGATGGATGGCCTTTGTACCTAGCAACGTAATTTACCGGAATTGGTTGATGTAAATTTTACCTTCTCCATGCGTGTCTTCCGTATTCCTTGCCCTGTTGTAGTTATGCTTGAT |
| P*enoA* | CTACCCCACCATCCCACTATTTTTGTTACGGTAGCCATGACCCCTCCATGGCAAAGAGAGAGGAGGACGAGGACGATCAGGAAACTGTGTCTCGCCGTCATACCACAATCGTGTTATCCTGATTGACATCTTCTTAAATATCGTTGTAACTGTTCCTGACTCTCGGTCAACTGAAATTGGATCTCCCCACCACTGCCTCTACCTTGTACTCCGTGACTGAACCATCCGATCATTCTTTTTGGGTCGTCGGTGAACACAACCCCCGCTGCTAGTCTCCTTCCAACACCGATCCAGAATTGTTTTGATTTTCCATTCCCTTCGTTTATATCTGTCGTCTCTCCTCCCTTTCCGTCTCTTTTCTTCCGTCCTCCAAGTTAGTCGACTGACCAATTCCGCAGCTCGTCAAA |
| T*agdA* | TGAAGGAAGCGTAACAGGATAGCCTAGACCCACATACTATCTGTATACAACTCCGCAATATGAAGTGATGAATGCAAACTAGCAGCGAATCGGATATCAGTAGCATAACGTAATCGGTAAGCGAGTTGCCCGCGCAAGCGAGTTGCCCACCACACGCGTTTTCAACGCGCCTCAATTTCTTAGATGATTAAAACATCAGCCATACCACCAAAAATACCTAAATCAAAAAAATCACGCGTTGAGCAGGAAGGCAGAATCCTATTAGCTATATCAGCTATAAAAAAACAAGAAATCAGCAGTTTTAGAAAGGCAGCTGAAATTTTTAATATACCTATCGCTACACTACGTTATCGTCTAAATGGAGGTTCCTTTCGAAATGATACTCGTGCCAATAGTTATAAAATAACTTCTAGTGAAGAGAAATCGCTTAAAAAATAGATTCTATCACTAGATAAACGTGGAGCACCTCCTCGGCCTGTACACGTACGAGAAATAGCCAATATCCTGCTTTTAAAGCGTAATACTACCTCCCCCCCTACTACTGTAGAGGAGAAATGGGTATACAACTTTACCAAGCGTACACCTAAGCTTAAATTCTGCTTTGCACGTCGCTACAACTATCAGCATGCCAAGGTAGAGGATCCTAAGGTTCTAGGTACTTAGTTTAAGCAGGTAAATAAGGTTATTCAGAAGTACGGTATAGCTTCAAGCGATATATACAATTTTAATAAAACGGGGTTTATAATGGGCCTAATAGCTACAGCCAAAGTTGTTACTAGATCTAATATGCCAGGGAAACTATTTTTATTACAGCTAGAGAACCGGGAATGGGTTACTGCC |
| P*amyB* | AATTCATGGTGTTTTGATCATTTTAAATTTTTATATGGCGGGTGGTGGGCAACTCGCTTGCGCGGGCAACTCGCTTACCGATTACGTTAGGGCTGATATTTACGTAAAAATCGTCAAGGGATGCAAGACCAAAGTAGTAAAACCCCGGAGTCAACAGCATCCAAGCCCAAGTCCTTCACGGAGAAACCCCAGCGTCCACATCACGAGCGAAGGACCACCTCTAGGCATCGGACGCACCATCCAATTAGAAGCAGCAAAGCGAAACAGCCCAAGAAAAAGGTCGGCCCGTCGGCCTTTTCTGCAACGCTGATCACGGGCAGCGATCCAACCAACACCCTCCAGAGTGACTAGGGGCGGAAATTTAAAGGGATTAATTTCCACTCAACCACAAATCACAGTCGTCCCCGGTATTGTCCTGCAGAATGCAATTTAAACTCTTCTGCGAATCGCTTGGATTCCCCGCCCCTGGCCGTAGAGCTTAAAGTATGTCCCTTGTCGATGCGATGTATCACAACATATAAATACTAGCAAGGGATGCCATGCTTGGAGGATAGCAACCGACAACATCACATCAAGCTCTCCCTTCTCTGAACAATAAACCCCACAGAAGGCATTT |
| P*xyrA* | CTGGCATAGGAGTTTACGGGGAGGGATACTGGAGCATGCTCTGCGGGGGACTTATTGGTTTTTCTCAACTTTGTGATGTACTTTTAGTCTGATACCAATTTTGTAGTTATACACAATATGGATCTAATGTCTGTTTCATACTTACTACTTGTTCCTTGAGTAATTGGTGGGATTTGGGTCCCGTGATGGATCCAACACGGACAGTGACCAATTGGGTTTGGCGCTTCTCTACATTTACCCGGCGACTCTCATTTAGCCGGAATGGGATCTTGTTGAGCGCCACTTTAGCCTCAAGACAGGGACATTCCTCCTCGTGTGACTGGCCTTTTCGGCCTAGCTGGCCTGGATCACGTCGCATTTATCCCGAGTGGGTATGGCCGTACCCCAAATGAGTGCTTTTTGGAAGAATATGCGCGGGGGCAATCTCCAAAAGTTGATTTATgcccctccttttctttgcggATACTTCTCTCATTTCTTTAACACTCTTTACTCTCCTCTCAACTGCAGTCGCTCTTCTCATTAAGCGCAATCTCTACACACAATAATCACA |
| *pks17* | ATGGAAGGCCCCGGTCATGTATATCTCTTCGGAGATCAGACTGCGGAGTTTGACTCGGGcCTTCGTCGTCTGCTGCATGCTAAGAATGACTCGCTGCTAGCAGCATTCTTCCAAAAGTCTTACTATGCGTTGCGCAAGGAAATTACATCGCTTCCACCATCTGAACGTCAGGGCTTCCCTCGTTTTACCAGTATCGTTGATCTCCTCGCGAGGTTCAAAGAGTCAGGGCCAAACCCAGCTCTAGAGAGCGCATTGACGACAATTTACCAATTGGGGTGTTTCATCCAGTAAGCATATTACCGAATCTTTGATAAAGAGACTATATTTGGAACCATATTTACTGACAATTTTTATAGCTACTATGGTGATTTGGGCCATGCCTATCCGTCAGGTGATGAGAGCTGCATCATAGGTCTTTGCACAGGTCAGCTCGCATCTGCAGCTGTGAGCTCTTCGAGGACTATCGGCGAGCTCATCCCGGCTGGAGTTGAGACCGTTATTGTTGCCTTGAGACTCGGCATGTGCGTTTTGAAAGTACAAGAGCTCATAGAACCTAGCAAATCGGCAACACCAAGCTGGTCTGTGCTAATTTCCGGCATGCGCGAGGCCGAGGCTCAGGATTTGATTCAGCAATACGCGGAGAAGAATGTAAGTCTCTTCCCCCAAACCTGCTTTCAATAAAATGGGATTATTGTTAATATTTCAAAGGCTTTGCCCCGGGTTTCTCAGCCTTATATAAGCGCAGTCAGCCCTAATGGACTCACGATCAGCGGTCCTCCCACTTTCCTAGGCCGTTTCATTGAGGCCAGTCTCTCGAAGGAGCACAAGCCTACAAGAGTTCCCATCCACGGCCCTTACCATGCTTCGCATTTGTATGATGAGAGGGATATTAACCGAATTTTGGAGTCTTGGCCTACCGAACAGTTCGTGAACTACGTGCCTCAGATTCCTGTTCTGTCAAGCGAGACAGGCAACGAATTCCAAGCCGAGAGTCTTGACCAGCTCTTGAGATCTTCTCTCCAGGAGATTCTGCAACGACAGCTCTGCTGGGACAAGGTCATCGAATCTTGTCAGTCCACCTTGGAAGCAGCTACAACATGCACTCTGTTCCCAATTTCCAGCACTGCAACCCAGAGTCTATTCAACTCACTGAAGAAGGCTGGAGTCTCCAATATCGAGGTTGACAGCACAATTGGTGATGTCCAGAAGGACTCTGAAGGAGACAACCGTACTGGCCGTTCTGAGAAATCTAAGATTGCAATCATTGGTCTTTCAGGGAGATTCCCAGAGTCACCTGACACCGAAGCATTCTGGGATCTTTTGAAAAAGGGGCTGGACGTTCACCGTGAGGTCCCCCCAGAGCGATGGGATGTCAAGGCTCACGTCGACATGGAAGGAAAGACAAGAAACACCAGCCAAGTTCAATACGGATGCTGGTATAACGACGCCGGAATGTTTGACCCTCGATTCTTCAATATGTCTCCCCGTGAAGCGCTTCAAGCAGACCCTGCTCAACGTCTGGCACTTCTTACGGCTTATGAGGCGTTGGAAATGGCGGGATTCATCCCAGACAGCACGCCCTCTACGCAAAAGAACCGTGTGGGTGTCTTCTATGGAATGACCAGCGATGATTACCGTGAGATCAACAGTGGTCAGGACATTGACACCTACTTTATCCCGGGTGGTAACCGTGCTTTCACTCCTGGTCGCATCAACTACTATTTCAAGTTCAGCGGCCCTAGTGTCAGTGTGGATACAGCTTGTTCCTCGAGTCTTGCAGCAATTCATGTCGCCTGTAATTCCCTGTGGAGGAATGAATGTGACTCTGCCGTCGCCGGTGGCGTCAATATCCTGACAAATCCTGACAACCATGCGGGCCTTGACCGTGGACACTTCCTCTCAAGGACTGGAAACTGTACCACCTTTGATGATGGGGCTGATGGCTATTGCAGAGCAGATGGAATTGGCTCCATTATCATTAAGAGACTCGAAGATGCTCAGGCAGACAACGATCCCATCTACGGTGTCATTGGAGGAGCTTACACCAACCACTCCGCAGAGGCTGTATCAATTACGCGGCCTCATGTAGGAGCACAGTCCTTCATCTTTGACAAGCTTCTCAACGAGTCCAACAGCGACCCGAAGGAGATCAGCTATATTGAAATGCACGGAACTGGCACCCAGGCAGGTGATGCAGTGGAGATGCAGTCGGTTCTCGATATCTTCGCTCCTGATTACCGTCGTGGACCGACTCAATCTCTTCACCTCGGTTCTGCAAAGTCAAACGTTGGCCACGGAGAGTCCGCCTCTGGTGTCACAGCTTTAATCAAGGTCTTGATGATGATGCGGAAGAACATGATTCCACCTCACTGCGGAATTAAGACCAAGATCAACCACAACTTCCCGACAGACTTCCCGCAGCGCAACGTGCACATTGCGTCGGAGCCTACCCCCTGGAACAGGCCAAATGGGGGCAAGCGGAAGACTTTCGTCAACAATTTCTCGGCTGCCGGTGGTAATACTGCTCTGATGGTCGAGGACGGTCCCCTTAATGAGGAGAATGTGGAAGACTCCCGGTCTGCCCATCCTGTGCTTGTCTCTGCTCGCTCTCAGTCTGCGCTGAAGAACAACATCTCTGCCCTCGTGCAGTACATAGACACAAACAAGAACTCGTTCAATACCAACGAGGCTAGCCTTCTTGCCAATCTGTCTTACACAACTACGGCCAGGCGCATTCATCATCCCTTCAGAGTCGCTGTCACTGGATCGACTCTGGATGAAGTCAGGAGTGGATTGGCTCCTATCGTCAACCGAGACAGCATAAGCCCGGCTCCTGCCAATCCACCTGGCATTGGCTTCGTTTTTACCGGACAGGGTGCTCAATACGCAGGCATGGGACGCCAATTGTTTGAGAGTTGCTCCCAATTCCGTGCCCACATTGAGCATTTGAATTGCATTGGTCAGAGCCAGGGCTTCCCATCTATTTTGTCCTTAGTCGATGGAAGTGTGCCAATTGAAGAGCATAGCCCGGTCGTTACCCAGCTTGGAACAACCTGTGTGCAGATGGCATTGACTAAGTACTGGATGTCTTTGGGTGTCTCCCCTGACTTTGTCATGGGCCATAGCCTCGGCGAGTTTGCCGCACTGAATGCTTCCGGTGTCTTGACGATCAGTGACACAATCTACCTTGCCGGGCGACGAGCCCAACTTCTCACCGAGCGAATAAAGGTTGGAACACATGCTATGCTTGCTATCAAGTCTTCAGTAGCACAGGTCAAGCAGTTCCTTGATGAGGCTACCGAGGTCGCTTGTATCAATGCGCCCAGCGAGACTGTCATCAGTGGCGCATGCGAGAAAATCGACGAGCTTGCACAAGCTCTTACGAACGAAGGTTTCAAGACTACCAAGCTGAGTGTACCTTTCGCTTTCCATTCAGCCCAGGTTGAACCTATTCTCGAGAGTCTTGCCGAAATTGGAAAAGGTGTCACTTTCAATATGCCCTCCATTCCATTCGTATCGGCCCTTCTTGGCGATGTCATCAATGAGACCAACAGCGAGGTGCTTGGACCCAACTACCTCACCCGCCACTGCCGGGAGACTGTTAATTTCCTGGGTGCTCTGGAAGCTACCCGGCACTCTAGCCTGATCAACGACAAGACAATCTGGGTTGAGATTGGCTCTCACCCCGTCTGCTCTGGGATGGTTAAATCTACTTTCGGTCCTCAGGCAACTACTGTGGCATCTCTCCGTCGCCAGGACGATACTTGGAAGGTTCTCTCGAATAGTACTTCCGCTCTTTATCTGGCTGGGATTGAACTTCGATGGAAGGAGTATCACCAGGACTTCACTGCCGGACACAAAGTCCTTCCACTGCCTTCCTACAAGTGGGATCTTAAGAACTATTGGATTCCCTACACTAATAACTTCTGTCTGCTCAAGGGTGCACCGGCGGCAGCAGTAGCTGAGGCTGCGCCAGTCGCTGTGTTCTTGTCATCAGCAGCCCAGAGGGTTTTGGAGACGAGTGGTGACAATTCGTCGGCATCTATCGTCATCGAGAATGACATTGCTGATCCCGACCTCAATCGCGTCATTGCAGGTCACAAGGTAAACGGTGCCTGTCTTACTCCATCGGTATGCCTATTTCCAATTTCCCACATAACAGTCTTGATTGCTAACTAAGTCTTAGTCCCTGTACGCCGATATTGCGCAAACACTCGGTGAATATCTAGTTCAGAATTACAACCCTGAGTGGAAAGATCGCGGGTTTGATGTTTGCAACGTGGTGGTCCCCAAGCCGCTTATTGCGAAGGGAGGCAAGCAACTCTTCCGCGTCTCAGCCACCGCAAATTGGGCCGAAGAAAATGCCAAGGTACAGGTGTGGTCTGTGACTCCGGAGGGGAAGAAGATTCTTaACCACGCTACTTGCGACATCAAGTTCTTTGATCCCAGTGCTTACGAGAGCGAGTGGAAGCGCAGCTCTTACCTGATCAAGCGAAGCATTGAGCATCTCCAGGAGAGCACGATATCCGGCCAGGCTCATCGCATGAAGAGAGGAATGGTCTACAAGCTCTTTGCCTCTCTGGTCGATTATGACGACAACTACAAATCCATTCGGGAAGTCATCCTCGACAGTGAACAACATGAGGCTACCGCGCTGGTCAAGTTCGAGGCTCCGCCGGGCAACTTCCATCGCAATCCATTCTGGATTGACAGCATCGGTCACTTGTCTGGCTTCATTATGAACGCCAGTGATAATACTGATTCGAAGAACCAAGTGTTCGTCAATCATGGATGGGACTCGATGCGGTGCTTGAAGAAGTTCGATCCCAGTGTCACTTACCGTACTTACGTGAGGATGCAGCCCTGGAAAGACTCTATCTGGGCTGGTGATGTCTATATGTTTGAAGGTGATGACATCGTTGCTGTGTATGGTGGTGTTAAGGTATGTTGAAAACCCGTCCGACGATTCTCGAGTGAATCACTAACAGAATGCGATATAGTTCCAAGCTCTGGCACGGAAGATCCTCGACATGGCTCTTCCTCCTGCTGGAGCCCCGGCACCCAAGCCAGCCGCCAAGCGCGTTCCTGCTCCGGTCGATATCCAGAAGGCAAAGACAGGCCCTGCCAAAAAGGCTTCTCCTTCACCAAAGTCCGGATTGCCAAGTTTGGCTACGCGCGCCCTTGCAATCCTGGCCGAAGAGGTCGGACTTTCGGCGTCTGAGATGACTGATGATCTCAACTTCGCTGATTACGGAGTTGACTCGCTGCTCTCGCTGACTGTCACTGGGCGATACCGTGAGGACATGGGACTCGATCTTGACTCCAGTGTCTTTGTGGATAATCCAACTGTCAAAGCTTTCAAAGGTCTCCTAGCTCAAATGGGCCCTAGCGAAAGTAGCGATGGATCAAGCAGCGAAGGTGACATGTCGTCGGCCGCGTCCACGACCGACATGTCGTCTCCAAACACCAGTGGTCTGCCAACTCCTGCAAATGAGAAGTCTATGACACACGGATTGCAGGAGCAGAATGACAGCATGAGGCAGATTGCCTCAATCTTGGCCGAGGAGATTGGCGTTGAGCCTGAGGAGCTCAGTGGCGATGCGAACCTTGGTGAGATGGGTCTGGACTCATTGATGTCGCTCACAGTCCTCGGTAAGATCCGTGAAGATATCGATCTCGATCTTCCTGGAGAGTTCTTCATTGAGAATCAGACACTCGATGATATCGAGACTACCCTGGATCTGAAGCCTAAGCTCGCTCCCGCGGAGCCAATCAGACTCCCCGAACAAATCCCAGCTCAAGCCCCGGTGGTCGCCCCCAGAACTGCCGTCCAGCACCCGCCGGCCACTTCGATCCTCCTGCAAGGAAACCCCAAGACAGCGAGTCAAAAGTTGTTCTTGTTCCCTGATGGCTCTGGTTCCGCCACCTCCTACGCTACAATCCCTGGAATTTCCACCGATGTGTGTGTATACGGACTGAACTGCCCATACATGCGCACCCCCGAGAACCTCAAGTTCAGCCTGGACGAGCTCACAGCTCCTTACGTTGCTGAGATCCGGCGCCGCCAGCCCACTGGCCCTTACAACTTCGGTGGCTGGTCTGCCGGTGGTATTTGCGCCTACGACGCCGCACGCCAGTTGATCTTCGAGGAAGGCGAGCGAGTCGAGCGCCTGCTCCTCCTAGACTCACCCTTCCCCATCGGGCTGGAGAAACTGCCTCCCCGTCTGTACAGCTTCTTCGACACGATCGGACTGTTTGGGGAGGGCAAGGCACCTCCACCCAAATGGCTTCTTCCTCACTTCCTGGCCTTCATCGACTCCTTGGATGCTTACAAGGCAGTTCCATTCCCTCACACAGACCCCAAGATGGCCGACAAACTTCCTAAGACCTTCATGGTCTGGGCCAAGGATGGTGTCTGCTCTAAGCCAGGTGATGCCCGCCCTGCACCAGCTGAGGATGGTAGCGCAGACCCCCGTGAGATGCTCTGGCTGTTGAACAACCGCACTGACCTCGGTCCGAATGGTTGGGATACCCTTGTCGGGCCGAAGCATGTCGGCGGTATTACCGTTATGGAAGATGCAAACCACTTCACTATGACCCGTGGCGAGAAGGCTAAGGAGTTGGCTGCATTCATTGCTAACTCTATGGCTTCTGTGTAA |


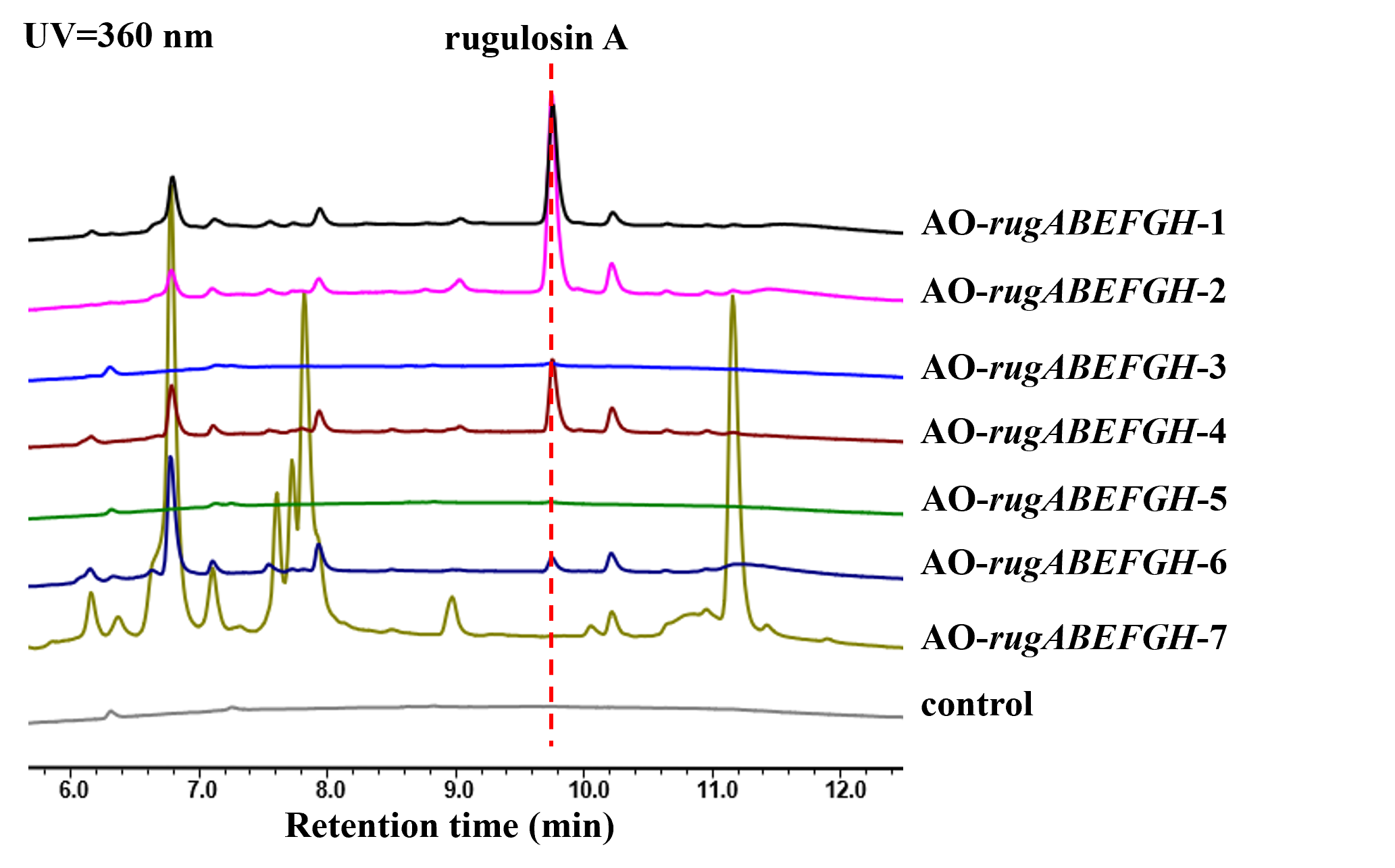


**Fig. S1** The chromatogram profiles of metabolites produced by *rug* transformants. Mycelia of transformants grown on CD agar plates were resuspended in 300 µL methanol and the supernatant dealt with 0.22 µm filter was subjected to LC-MS detection.

[M+H]^+^:

[M-H]^-^:

**Fig. S2** The MS spectrum of rugulosin A produced by AO-*rugABEFGH*-1. Rugulosin A: C_30_H_22_O_10_, [M+H]^+^ calculated 543.1286, observed 543.35; [M-H]^-^ calculated 541.1140, observed 541.15.
